# Understanding the clinical management of obstructive sleep apnoea in tetraplegia: a qualitative study using the theoretical domains framework

**DOI:** 10.1186/s12913-019-4197-8

**Published:** 2019-06-21

**Authors:** Marnie Graco, David J. Berlowitz, Sally E. Green

**Affiliations:** 1 Institute for Breathing and Sleep, Austin Health, Melbourne, Victoria Australia; 2 Department of Medicine, The University of Melbourne, Melbourne, Victoria Australia; 3 Department of Physiotherapy, The University of Melbourne, Melbourne, Victoria Australia; 4 School of Public Health and Preventive Medicine, Monash University, Melbourne, Victoria Australia

**Keywords:** Sleep apnea syndromes, Spinal cord injuries, Theoretical domains framework, Semi-structured interviews, Qualitative research

## Abstract

**Background:**

Clinical practice guidelines recommend further testing for people with tetraplegia and signs and symptoms of obstructive sleep apnoea (OSA), followed by treatment with positive airway pressure therapy. Little is known about how clinicians manage OSA in tetraplegia. The theoretical domains framework (TDF) is commonly used to identify determinants of clinical behaviours. This study aimed to describe OSA management practices in tetraplegia, and to explore factors influencing clinical practice.

**Methods:**

Semi-structured interviews were conducted with 20 specialist doctors managing people with tetraplegia from spinal units in Europe, UK, Canada, USA, Australia and New Zealand. Interviews were audiotaped for verbatim transcription. OSA management was divided into screening, diagnosis and treatment components for inpatient and outpatient services, allowing common practices to be categorised. Data were thematically coded to the 12 constructs of the TDF. Common beliefs were identified and comparisons were made between participants reporting different practices.

**Results:**

Routine screening for OSA signs and symptoms was reported by 10 (50%) doctors in inpatient settings and eight (40%) in outpatient clinics. Doctors commonly referred to sleep specialists for OSA diagnosis (9/20 in inpatients; 16/20 in outpatients), and treatment (12/20, 17/20). Three doctors reported their three spinal units were managing non-complicated OSA internally, without referral to sleep specialists. Ten belief statements representing six domains of the TDF were generated about screening. Lack of time and support staff (*Environmental context and resources)* and no prompts to screen for OSA *(Memory, attention and decision processes)* were commonly identified barriers to routine screening. Ten belief statements representing six TDF domains were generated for diagnosis and treatment behaviours*.* Common barriers to independent management practices were lack of skills *(Skills)*, low confidence (*Beliefs about capabilities)*, and the belief that OSA management was outside their scope of practice *(Social/Professional role and identity)*. The three units independently managing OSA were well resourced with multidisciplinary involvement *(Environmental context and resources)*, had ‘clinical champions’ to lead the program *(Social influences)*.

**Conclusion:**

Clinical management of OSA in tetraplegia is highly varied. Several influences on OSA management within spinal units have been identified, facilitating the development of future interventions aiming to improve clinical practice.

**Electronic supplementary material:**

The online version of this article (10.1186/s12913-019-4197-8) contains supplementary material, which is available to authorized users.

## Background

People with tetraplegia experience a range of complications from their injury, affecting almost every system of their body. Obstructive sleep apnoea (OSA) is one such complication, with prevalence estimates of up to 83% in the acute phase, and up to 97% in the community dwelling chronic population [1, 2]. The quality of life of people with tetraplegia and OSA is up to five times the minimally important clinical difference worse than their peers without OSA [3]. OSA has been associated with daytime sleepiness, poor memory, attention and information processing in both the acute and chronic populations, and is therefore likely to impact on rehabilitation and vocational outcomes [4, 5]. Improving the management of OSA has the potential to prevent these undesirable consequences of spinal cord injury (SCI). A recent multicentre randomised controlled trial of treating CPAP following acute, traumatic tetraplegia found that while CPAP did not improve neurocognitive function, it did improve subjective daytime sleepiness [6].

Guidelines developed by the Consortium of Spinal Cord Medicine recommend diagnostic testing with polysomnography for all people with SCI with excessive daytime sleepiness or other symptoms of sleep disordered breathing [7]. These guidelines also recommend the prescription of positive airway pressure (PAP) therapy for those with a positive diagnosis of OSA. Similar recommendations have been published by the Spinal Cord Injury Rehabilitation Evidence (SCIRE) project, a Canadian research collaboration that produces evidence-based practice recommendations for health professionals working in SCI rehabilitation [8]. The SCIRE recommendations include vigilance for suggestive signs and symptoms and further testing with oximetry or polysomnography when these signs are present. Management adherent to these recommendations therefore requires routine screening for the signs and symptoms of OSA, and subsequent investigation.

Both guidelines are not explicit in fully detailing the recommended clinical practices, potentially hampering efforts by clinicians aiming to practice according to evidence-based guidelines [9]. In particular, screening practices are recommended with little indication of how, when or where screening for signs and symptoms of OSA should be undertaken. Diagnosis of OSA is recommended with polysomnography in one guideline, and polysomnography or oximetry in the other, with no indication of who would perform these tests and what the clinical criteria for diagnosis should be. Furthermore, only one guideline recommends a specific type of treatment; initially with continuous positive airway pressure (CPAP), and with bi-level PAP as a second option for those unable to tolerate CPAP.

The lack of actionable recommendations in the OSA in SCI guidelines reflects a lack of robust clinical evidence. While the guidelines are based on evidence from non-randomised studies and the expert panel consensus was reported to be strong, there is little randomised trial evidence in this setting. SCI is a relatively small and specialised clinical area, and as such, there are significant challenges for the conduct of clinical trials [10]. Thus, few guidelines in SCI are based on strong evidence. A review of knowledge translation research in SCI revealed almost all interventions were based on the findings of individual studies and expert opinion, with only one citing evidence from a randomised control trial [11]. Given the high prevalence and significant morbidity of OSA in tetraplegia, practice concordant with the best available evidence in the form of the current guidelines is important, and will contribute to reducing variation in practice and improving the clinical management of OSA.

Very little is known about the current management of OSA in chronic tetraplegia. An older study investigating OSA treatment in people with chronic SCI found that in a service providing care to approximately 600 veterans with chronic SCI, approximately 15% of people with tetraplegia had received a diagnoses of OSA [12]. Given the high prevalence estimates in this population, this is likely to reflect low screening and subsequent testing for OSA. More current research is required to determine the extent of OSA under-diagnosis in the present clinical environment.

To our knowledge, there have been no studies that systematically describe the current management of OSA in SCI, nor what influences the clinical behaviours of health professionals involved in the care of people with SCI and OSA. Anecdotally, practice is highly varied. A systematic review of barriers to physician adherence to clinical practice guidelines generally (not specifically in SCI) identified many factors that may influence practice, including lack of awareness, familiarity and dis/agreement with the guidelines, poor physician self-efficacy, low outcome expectancy, inertia of previous practice and external barriers such as lack of time, environmental factors and staff shortages [13]. Understanding the prevailing and contextual influences on clinical practice is essential for the development of any intervention aiming to improve the management of OSA in people with tetraplegia.

The Theoretical Domains Framework (TDF) is a validated and commonly used set of 12 behavioural domains for use when exploring factors that influence clinical behaviours [14]. The 12 domains of the TDF include: knowledge; skills; social/professional role and identity; beliefs about capabilities; beliefs about consequences; motivation and goals; nature of the behaviour; memory, attention and decision processes; environmental context and resources; social influences; emotion; and behavioural regulation. The TDF enables a comprehensive, theory-based approach to recognising the behaviours that need to be changed, thereby identifying opportunities for improved practice.

The aims of this study are: 1. To describe the OSA screening, diagnosis and treatment practices of specialist doctors managing the rehabilitation of people with tetraplegia. 2. To explore factors that influence the management of OSA in tetraplegia, informed by the Theoretical Domains Framework.

## Method

In-depth semi-structured interviews were conducted with specialist doctors managing the rehabilitation of people with tetraplegia in the inpatient and outpatient settings in SCI rehabilitation centres between August 2016 and March 2018. Initially names and contact details of doctors were obtained from websites of hospitals with a specialised SCI Unit. Additionally, a snowball sampling technique, where existing participants recommended future participants from among their professional networks, was utilized. In recognition that practice may vary in different regions because of cultural and healthcare model influences, a purposeful sample was drawn to include a range of regions in the Organisation for Economic Co-operation and Development (OECD; e.g. Australia/New Zealand, North America, Europe, UK). Low and middle-income countries were not included because the availability of resources and infrastructure required to undertake OSA management, and hence culture and subsequent practice, were likely to be very different. Potential participants were approached by telephone or email and invited to participate in the study.

Of the 25 doctors approached, five doctors did not respond to email; none officially declined the invitation to participate. Ethical approval for the study was obtained from the University of Melbourne (School of Health Sciences Human Ethics Advisory Group; Ethics ID 1545475). The University of Melbourne ethics committee provided ethical approval for recruitment of doctors from overseas because of the low-risk nature of the study, and the practical implications of obtaining ethics from multiple countries for the recruitment of small numbers of health professional participants (ie one doctor per site). Further, the participant recruitment strategy detailed above precluded us from pre-emptively knowing which countries we would recruit from and prospectively apply for ethical approval from each country. All participants provided written, informed consent prior to the interview.

Interviews were conducted face to face or via online video technology (e.g. Skype) or telephone at a time suitable to the participants. The researcher conducting the interviews (MG) had experience with qualitative research methods and the clinical area. An interview schedule, based on the TDF, was used to prompt the discussion and guide the analysis (see Additional file 1). The interview questions focussed on describing current practices in the identification and management of OSA, and exploring the domains of the TDF to understand factors that influence practices. See Additional file 1 for interview guide.

**Fig. 1:**
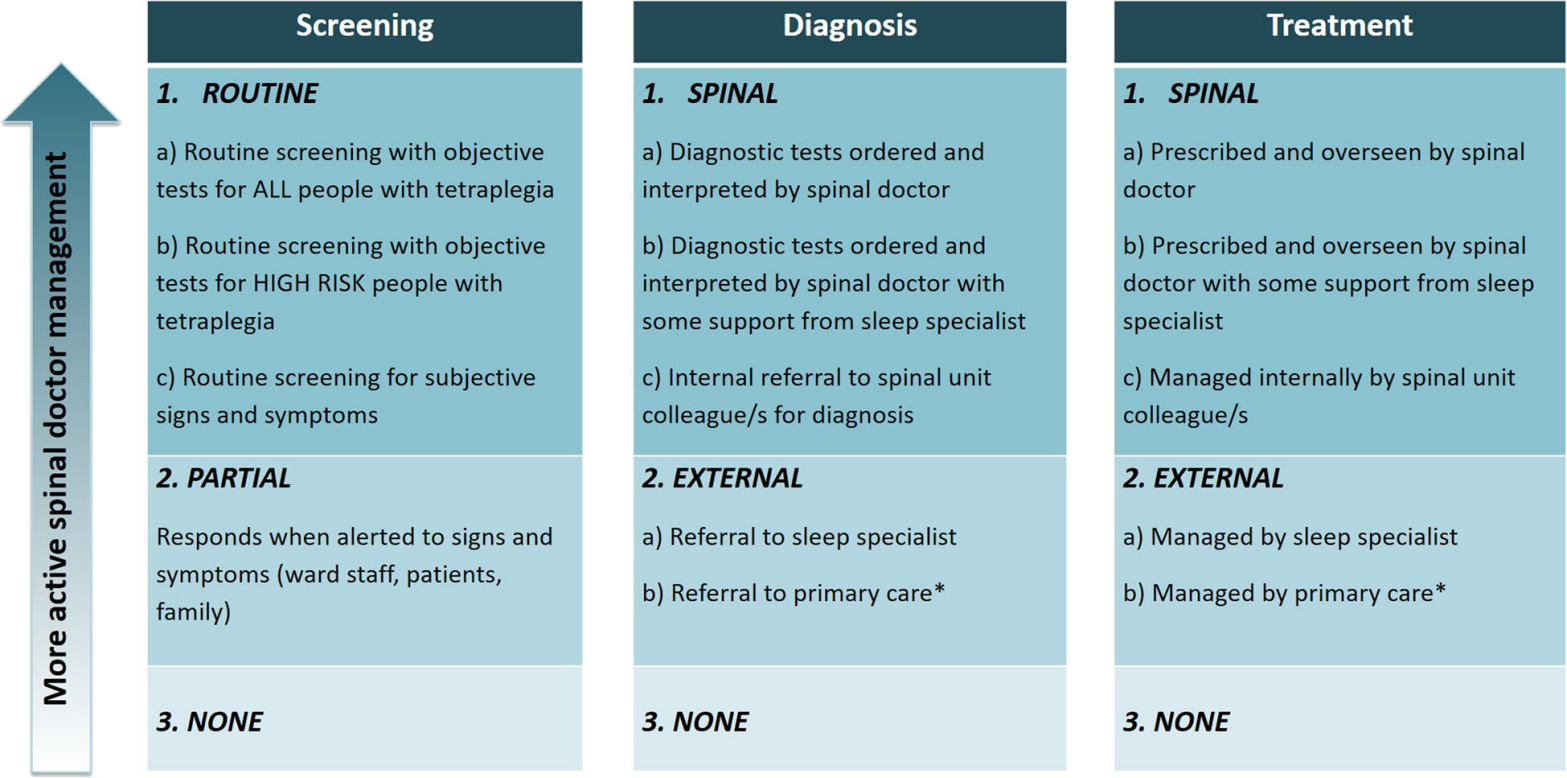
Predominant clinical practices. *outpatient clinical management only

All interviews were audio recorded for verbatim transcription. Transcripts were de-identified and imported into NVivo qualitative data analysis software (QSR International Pty Ltd. Version 12, 2018) to aid data management and analysis. The OSA management pathway was divided into screening, diagnosis and treatment components for inpatient and outpatient services, creating six categories. Self-reported practice was initially content analysed into these six categories. Data from the first five interviews were then independently analysed by two researchers (MG and DJB) to identify common clinical practices within each of the categories. For example, for inpatient diagnosis, clinical practice was found to fall within three main clinical practices. They were: *conducted by spinal unit* (with three sub-clinical practices), *referral to sleep specialist* and *not undertaken*. After the first five interviews, the two researchers discussed and resolved any differences in their identified clinical practices. Some changes to wording were required to ensure consistency and clarity. The clinical practice categories are presented in Fig. 1.

This matrix formed the coding structure for the remaining interviews, and as such, six clinical practice categories were assigned for each participant. When clinicians reported more than one clinical practice (e.g. referring to sleep specialist and general practitioner in outpatients), the predominant practice was selected.

Data were next thematically coded according to the 12 constructs of the TDF to assess influences on clinical practice and develop theoretical explanations about the influences on practice. The first five interviews were analysed independently by two researchers (MG and DJB). MG and DJB met after coding two interviews, and again after five, to discuss and resolve any differences and to revise the coding guideline. The remaining interviews were analysed by MG, who discussed any instances of ambiguity with DJB.

Following coding to the TDF domains, common “belief statements” were generated. A belief statement has been defined as a collection of responses with a similar core belief about the barrier or enabler to the behaviour under investigation [15]. Comparisons in belief statements were made between participants with different clinical practices. For example, the influences identified for those routinely screening all their patients with tetraplegia were compared to those who were not. Similarly, the influences of those who were diagnosing and/or prescribing treatment for OSA themselves were compared to those who were referring to sleep specialists for OSA diagnosis and treatment.

After the first five interviews were analysed, sampling continued until saturation of themes was achieved. Saturation was defined as when five consecutive interviews had been analysed with no new belief statements emerging [16].

## Results

Interviews were conducted with 20 doctors from 20 spinal units. All doctors were employed as specialist rehabilitation consultants in stand-alone specialist SCI rehabilitation units or general rehabilitation units with substantive SCI caseloads. All had at least five years experience practicing in SCI rehabilitation medicine. Two doctors had initially trained as surgeons while 18 had completed training in physical medicine and rehabilitation. Nine of the doctors were women. Specialist sleep laboratories were on site for six doctors, available in nearby affiliated hospitals for seven, and not available for another seven. Five interviews were conducted face-to-face, 10 with online video technology, and five over telephone. Interviews ranged in length from 22 to 66 (average 41) minutes.

### Predominant clinical practices

Of the five interviews double-coded, there were three differences in categorization of clinical practices (6 practice types, 5 interviews = 30 cells), which were resolved on discussion. Table 1 summarises the self-reported clinical practices of the 20 doctors.

**Table 1:** Results of categorisation into common clinical practices

	Inpatient *N* (%)	Outpatient *N* (%)
Screening		
1. Routine	**10 (50%)**	**8 (40%)**
a Routine screening with objective tests for all people with tetraplegia	8 (40%)	0 (0%)
b Routine screening with objective tests for high risk people with tetraplegia	2 (10%)	0 (0%)
c Routine screening for subjective signs and symptoms	0 (0%)	8 (40%)
2. Partial Responds when alerted to signs and symptoms	**10 (50%)**	**12 (60%)**
3. None	**0 (0%)**	**0 (0%)**
Diagnosis		
1. Spinal	**10 (50%)**	**4 (20%)**
a Diagnostic tests ordered and interpreted by spinal doctor	5 (25%)	3 (15%)
b Diagnostic tests ordered and interpreted by spinal doctor with some support from sleep specialist	2 (10%)	0 (0%)
c Internal referral to spinal unit colleague/s for diagnosis	3 (15%)	1 (5%)
2. External	**9 (45%)**	**16 (80%)**
a Referral to sleep specialist	9 (45%)	13 (65%)
b Referral to primary care		3 (15%)
3. None	**1 (5%)**	**0 (0%)**
Treatment		
1. Spinal	**8 (40%)**	**3 (15%)**
a Prescribed and overseen by spinal doctor	4 (20%)	3 (15%)
b Prescribed and overseen by spinal doctor with some support from sleep specialist	1 (5%)	0 (0%)
c Managed internally by spinal unit colleague/s	3 (15%)	0 (0%)
2. External	**11 (55%)**	**17 (85%)**
a Managed by sleep specialist	11 (55%)	16 (80%)
b Managed by primary care		1 (5%)
3. None	**1 (5%)**	**0 (0%)**

In the inpatient unit, 10 (50%) of physicians reported routine screening for OSA, with eight of these screening all patients with tetraplegia using objective tests (e.g. overnight oximetry). Ten (50%) reported diagnosing OSA within the spinal unit. Three of these referred internally to their colleague(s) for diagnosis, and two were provided with some assistance from a sleep physician. Of the spinal units diagnosing OSA, most used polygraphy (8/10) with two relying solely on overnight oximetry. Eight (40%) reported the prescription of treatment for OSA occurred within their spinal unit, with four prescribing treatment independently of any sleep specialist. Of these, seven offered CPAP as first-line treatment, and one predominantly prescribed bi-level PAP.

In the outpatient environment, eight (40%) reported routine screening for OSA in all patients with tetraplegia, with questions about signs and symptoms. The remaining 12 (60%) did not consider screening for OSA unless alerted to signs and symptoms from patients and/or their carers. These doctors also estimated that less than 10% of their patients were identified at risk for OSA, requiring further investigations. Three (15%) reported responsibility for diagnosing OSA in their outpatients with tetraplegia, and one referred internally to a spinal unit colleague. Of those diagnosing OSA, two used polygraphy and one predominantly used overnight oximetry. Doctors diagnosing OSA also reported managing the treatment and follow-up. The remaining 16 (80%) referred to a sleep specialist or the primary care physician for OSA diagnosis and ongoing management.

In summary, three doctors (15%) reported that their spinal unit was predominantly diagnosing and treating non-complicated OSA in their inpatients and outpatients with tetraplegia. Eleven (55%) were predominantly referring all inpatients and outpatients with signs and symptoms of OSA to sleep specialists or GPs for diagnosis and management. The remaining six (30%) were practicing a “hybrid management model”; that is, predominantly diagnosing and treating OSA in their inpatient units, and referring to external specialists in their outpatient clinics.

### Factors influencing practice

For the qualitative analysis of factors influencing practice, OSA management practices were divided into *screening practices* and *diagnosis and treatment practices.* Diagnosis and treatment practices were not analysed separately as they were considerably related to one another. For example if a doctor referred for diagnosis of OSA, the referral also covered treatment. Similarly if a doctor diagnosed OSA, s/he tended to also prescribe the treatment. If the influences on clinical practice were specific to the inpatient or outpatient settings, these were clearly reflected in the belief statements.

### Factors influencing screening practices

Key themes regarding screening behaviours represented six domains of the TDF: Knowledge, Social/Professional role and identity, Beliefs about capabilities, Beliefs about consequences, Memory, attention and decision processes, and Environmental context and resources. Within these domains, 10 belief statements were generated, of which three were separated into opposite beliefs. Table 2 summarises the belief statements, corresponding TDF domains and representative quotes.

**Table 2:** Summary of relevant TDF domains, belief statements and representative quotes about screening for OSA in tetraplegia

Domain	Belief statements	Representative quotes	Frequency of belief out of 20
Knowledge	I don’t know of any clinical practice guidelines recommending management of OSA in tetraplegia.	“No I don’t know or aware of any existing clinical guidelines.”	10
		Regarding clinical practice guidelines: “I assume they [clinical practice guidelines] exist. But I wouldn’t go hunting for them because I don’t disagree with the concept that they should be screened.”	
	I know that the prevalence of OSA is very high in tetraplegia and that OSA causes negative outcomes.	“So the paper that I usually refer to…where they followed acute spinal cord injuries, so it was within the first year, and they test for sleep apnoea and it was up to like 80%. And then most other papers say, you know, up to 60% of spinal cord injury will have sleep apnoea.”	14
		“Yes. I’m aware it is high. It is definitely high in the first 2-3 months, but I can see a lot of the studies from one year post injury, that’s quite variable, it’s varies from 40-70%.”	
Social/Professional Role and Identity	As the doctor managing the patients’ rehabilitation and spinal cord injury needs, screening for OSA is my clinical responsibility.	“I think it should be the physician’s role. I think that’s the most appropriate person because if the symptoms come back positive, it does have to be a medical referral onto the respiratory clinic.”	17
		“I think it is our responsibility as their spinal cord injury doctor to understand sleep apnoea and understand respiratory; it falls under the umbrella of respiratory management, right. Especially somebody with a cervical injury, like you have to know what MIPS and MEPS are, vital capacities are, what their PFTs are. And sleep apnoea is just another component of that.”	
Beliefs about Capabilities	I am confident/not confident that I am identifying OSA in most of my patients.	“I think we get everything, we get all patients we need, well we catch all the patients who are in need of ventilation, yes.”	8
		“I’d say I’m pretty confident, yeah I don’t miss it in many patients.”	
		“I wouldn’t be very confident [to identify OSA symptoms]. The symptoms, there are so many other contributors to the symptoms that are described, I wouldn’t be very confident.”	8
		“In the acute phase, I think I’m probably missing a good proportion. Just ballparking, maybe 30%, 30 to 40%, I might be missing. In the community phase, of those that I follow regularly, probably missing less, but I’m sure I'm still missing some. Maybe 10%, 10–20%.”	
Beliefs about Consequences	Routine screening may identify non-symptomatic OSA that does not need to be treated.	“Okay, but even if you screen symptoms, and they have some symptoms, people can be affected by their symptoms in a different way. Did he have a problem? If he didn’t have a problem, why suddenly I found a problem with him and I start him to sleep with a machine on. The problem is blanket screening and blanket investigation we’ll end up having more people on a treatment that otherwise may not need to be. That is my worry.”	3
		“From my point of view, in the clinic, I’d probably be most interested in following up patients who had symptoms that were relevant to them. I guess a disincentive for me is to be actively pursuing investigation results of patients who don’t seem to have symptoms of that. Because what’s the point? I mean, like, with any test or referral, there’s a saying in medicine, don’t do it if it’s not going to change the treatment. Yeah, well it would be a waste of resources, but also it’s inconvenient for the patient.”	
	Routine screening helps prevent patients who are poor at recognizing their symptoms from being missed.	“Yeah because patients do not complain about that, that you have to measure it before you know that they have it, so sometimes they have the complaints of tiredness and that kind of stuff and then you have a trigger but if they don’t have that complaint then the screening might disappear.”	3
	I am/I am not sure that the benefits of routine screening would outweigh the costs.	“No question about it, yes. Because most patients, when they’re eventually getting ventilation during the night, they feel a lot better and they can have more… what do you call it, they can do much better during the day, so I think most patients will benefit from it (screening).”	13
		“Have to do it. Yeah, of course. The only long run if you ignore something which is there and you don’t treat it, you don’t manage it, of course at the end of the day that will cost you even more. And also you have to respect the patient’s wellbeing and their needs.”	
		“I wouldn’t be convinced. I’m not convinced of that at the moment, no. Should I just screen them all? I don’t know if that would be cost-effective, I don’t think so.”	5
		“I think it’s probably only worthwhile when the patient initiates the concern about fatigue and sleepiness because, otherwise, my experience is that if they’re really not troubled by symptoms in the day, they do not tolerate CPAP.”	
Memory, Attention and Decision Processes	A checklist/form is helpful/would be helpful to prompt me to screen for OSA in the inpatient unit and outpatient clinic.	“With our clinics we do have a template, we always get prompted to ask these questions about sleep, excessive snoring, does your partner notice you are not breathing for a while, and then we check the risk factors. So as long as the template is there we usually – I usually, you get prompted to ask it and I would.”	14
		“Inpatients definitely, so we have some standing orders ... And on there it was just immediate, everyone gets overnight oximetry and pulmonary function tests, and then in outpatient I do have like a template I use when I see patients, so there’s a respiratory heading which usually prompts me to ask about that.”	
		“And I often think, “Oh, gosh, I should remember to ask the patients about their breathing but I never seem to. So, I think that if there was a box, like, are you having sleep-disordered breathing symptoms, I mean, most doctors have an idea what those symptoms are, you could just quickly ask the patient four or five questions.”	
		“I think it will be nice if we can come up with a routine screen that we will screen everybody on admission, like an admission ASIA, something like that, we could do an admission and a discharge. If it’s a very short questionnaire that we can do. I think it would be worthwhile.”	
Environmental Context and Resources	I don't have enough time in outpatients to screen for OSA symptoms.	“I think it’s, for us like, probably the time that I am allotted with patients, so there’s a lot of things to cover.	6
		“In our current setup we don’t have time. We still allocate an hour for the patient, there are so many things to discuss, especially if they come once a year. And we don’t have any allied health clinic.”	
	Patients often have more important medical issues to discuss in their outpatient appointment than OSA.	“So they're having a very hard time with bladder, with bowel, with pain, spasticity, and then unfortunately the respiratory system does fall on the wayside a little bit. And if you – if they are really worried about their bladder, and you finish talking about their bladder, and they're thinking about their bladder, and start talking about sleep apnoea, they tend not to take it – it's hard to then take on so much information.”	6
		“Usually I’ll have the patient kind of lead the discussion as to what their most important thing they want to talk about that day is and I’ll kind of ask them prompting questions just to see a more general review of systems, but in that appointment, like, yeah I think that might be why things are getting missed because they may just want to talk about pain that day or they may just want to talk about their bladder or their pressure ulcer; we don’t get around to discussing sleep apnoea as well as we should.”	

Most doctors were not aware of any clinical practice guidelines about OSA management in tetraplegia, although they did know of research establishing high prevalence of OSA in tetraplegia (*Knowledge*). There was a strong belief that, as the person responsible for the holistic management of the person with SCI, screening for OSA was their responsibility *(Social/professional role and identity).* However many reported a lack of confidence in their ability to identify OSA in their patients. For some this was because they lacked confidence in identifying risk factors and symptoms, and for others, it was reflective of their incomplete screening practices *(Beliefs about capabilities).*

Some doctors were fundamentally opposed to routine screening for a condition that the patient may not recognise as a problem. In contrast, others felt that routine screening was important because patients often have difficulty recognizing their symptoms of OSA. Most thought that the benefits of routine screening would outweigh the costs. However some doctors were not convinced of the need for routine screening for OSA, which appeared to reflect a lack of confidence in the outcomes of the treatment. *(Beliefs about consequences).*

Those who reported routine screening practices for OSA tended to use reminders. These were usually a checklist or form in the outpatient clinic, standard orders for testing (e.g overnight oximetry) in the inpatient unit, or an agreed protocol for OSA screening within the spinal unit. Those who were not routinely screening for OSA, but believed that screening was their responsibility, commonly reported forgetting to screen in a busy clinical environment. When asked what they would change in their practice to improve the management of OSA, the most common response was the introduction of some sort of physical reminder, such as a form or checklist, to screen for likelihood of OSA (*Memory, attention and decision processes*).

Related to this was lack of time and resources, particularly in the outpatient environment, which was commonly cited as a barrier to screening. Nursing and allied health support were not available for most doctors in the outpatient clinic. Doctors spoke passionately about the patients’ competing medical problems, and the lack of available time to discuss all of their medical issues. Screening for signs and symptoms of a condition that the patient was not complaining about was not a priority when the patient had more significant medical problems such as bladder or bowel issues, pressure sores or pain. Most surveillance clinics for chronic spinal cord injury offered annual appointments. For one doctor, patients were reviewed twice yearly, enabling one of these visits to be dedicated to proactive screening for conditions such as OSA, while the other was focused on managing existing health problems (*Environmental context and resources*).

### Factors influencing diagnosis and treatment practices

Key themes regarding diagnosis and treatment behaviours emerged as representing six domains of the TDF: Skills, Social/Professional role and identity, Beliefs about capabilities, Beliefs about consequences, Environmental context and resources, and Social influences. Within these domains, 10 belief statements were generated, of which three could be divided into conflicting beliefs (Table 3).

**Table 3:** Summary of relevant TDF domains, belief statements and representative quotes about diagnosing and treating OSA in tetraplegia

Domain	Belief statement	Representative quotes	Frequency of belief out of 20
Skills	I don't have the necessary skills to interpret diagnostic tests and prescribe treatments for OSA.	“I don’t order oximetry or spirometry or something myself because I’m not sure how to interpret it.”	11
		“Lack of confidence and lack of training. Especially about the machines and about what pressures, and so on, to start with. I know that we would titrate it depending on the oximetry or the sleep study, but I would not know exactly how to start.”	
Social/Professional Role and Identity	The diagnosis and treatment of OSA is outside my scope of practice. It should be managed by a sleep/respiratory specialist.	“If I was looking up the literature that wouldn’t be something I’d look up because it would never be appropriate for me to be the one prescribing the treatment for sleep-disordered breathing.”	6
		“I don’t have the appropriate speciality qualification to interpret the results and prescribe the treatment. So, it would be sort of a, I’m trying to think of the word, it would be breaching my scope of practice. It would be implying to the patient that I know what I’m talking about when I don’t.”	
		“The way our system works is once I get pulmonologists involved it’s sort of like their thing.”	
		“I don’t consider myself a sleep specialist so if they’ve got symptoms that are consistent with that and there’s concerns on the oxygen saturation, that’s when I take them to the respirologist to see.”	
Beliefs about Capabilities	I am not confident to diagnose and treat OSA without sleep/respiratory specialist involvement.	“But I think I like having the respirologist there to discuss sort of a game plan of what pressures to start them at, even though it’s auto CPAP or, you know.”	12
		[Regarding diagnosing OSA] “I’ve not been trained in it. You know, I can read a graph but just because I can read labels I am not confidently able to say, “Yes, you have sleep apnoea.””	
		“Personally, I don’t feel confident in prescribing.”	
Beliefs about Consequences	CPAP is beneficial to my patients with tetraplegia and OSA.	“So once patients are diagnosed and treated successfully, the change in terms of cognitive improvement, we have patients who would sleep through their therapy sessions, their family meetings, because they were so tired. We have patients who are on numerous sleep inducers just to get them to sleep. So once we see that patients can come off of these medications, they’re fully participating and learning about their spinal cord injury, that’s huge, right, because that will decrease the length of stay in rehab, and all of the other complications associated with them.”	9
		“And then I’d say the more impressive thing that has happened, not uncommonly in patients who use it on our unit, is all of a sudden they do way better in tolerating therapies the next day, even day-to-day, like, “We’re going to try this tonight,” and the next day the therapists are like, “What did you do differently with Mr Smith? He’s like a different guy today.” It’s like, “Well, I think he has sleep apnoea and used CPAP last night. I guess his sleep apnoea was really affecting him.” And we have lots of the patients like that, I would say.”	
	Adherence to CPAP is poor/good in our unit.	“Of the patients who can’t take the mask off themselves, I'd say 80% of them don’t tolerate it. It’s bad but what are you going to do. I totally understand.”	7
		“I think the biggest challenge for us right now is to get people to adhere to the CPAP machine.”	
		“But patients just find it [CPAP] really difficult to tolerate, so most patients go untreated.”	4
		“No I would say normally we have a high compliance in tetraplegics… I would say 80% is compliant. We have of course some person who are not compliant and we check their compliance with the usual things.”	
Environmental Context and Resources	We have poor/good access to overnight sleep studies and sleep specialists.	“It’s hard to get an in-patient sleep study now…But, yeah, that’s been a bit of an inhibitory factor, you know, to ask about patients early on and then say, “Well, you can have a sleep study in 14 months when you’re out of hospital.””	6
		“It’s a logistical problem if they need a lot of care or ceiling lifts or anything like that, or an attendant. Because you know what sleep labs look like. They’re not designed for people in wheelchairs.”	
		“Having a sleep study is very difficult, for our inpatients, because [nearby acute hospital] has a sleep service but that is not manned, there is no nursing support.”	4
		“In a few weeks patients can go there and get the measurements, yep. And when we do it in our ward then it’s also very quickly, so the waiting list is no problem, no.”	
	I can’t diagnose OSA and/or prescribe treatment because the patient’s CPAP machine won’t be funded.	“But most commercial payers in [XX country] require that a polysomnography is done, documented before they’ll pay for it. So we’re kind of hamstrung a little bit in that way.”	7
		“I prescribe it, they won’t get funded. So there is a minority who can get funding or self-fund, but you still need to involve a respiratory professional in the set-up and reading and the compliance.	
	Our spinal unit has trained nurses and allied health to help manage OSA / We would need trained nurses and allied health to help manage OSA.	“Yeah we’ve got nurses involved in this part of our clinic. The nurses would go to the patients with our CPAPs and then advise them around the mask they would use and instruct them and all that.”	9
		“So, we use a couple of our physios that kind of are the respiratory leads but, actually, any of our physios have the competence to set up BiPAP, CPAP, etcetera.”	
		“We also need the nurses of course, they have to be knowledgeable about this, we have to train the team, the doctors, everybody else, so maybe in the future we will, yes.”	4
		“I need to have other special respiratory nurse who needs to train and they need to educate.”	
	I practice in the same way as my colleagues from the same spinal unit.	“We do the same thing. Whoever it is, they’ll be doing the same thing in our unit.”	16
		“I think we have a clear policy of all the screening and referring and intervention for sleep apnoea is probably standard practice.”	
Social influences	Our OSA management program is the result of a “clinical champion”	“It started with my colleague…maybe even 10 years ago or a bit longer he saw [another hospital’s] sleep laboratory and you know the screening on sleep apnoea they do in their spinal cord centre … my colleague got inspired and started to set up a similar department here which existed of nurses and himself and later I would take part in that as well and over the years kind of grew in our expertise I guess.”	6
		Participant: “You sort of need a champion.” Interviewer: “Right, so you’ve basically, you’re the one who set up this program for your unit?” Participant: “Yep, pretty much, yeah, yeah.”	
		“My colleague and I started 20 years ago and realised that our tetraplegic patients were falling asleep during therapies... And then, and then we started assessing our patients, realised this is a problem. And then since this experience done 20 years ago now and then it became the standard. It was just translation from research to daily routine and now it’s well implemented.”	

Those who were not diagnosing OSA or prescribing treatment did not believe they had the necessary skills or training to both interpret the diagnostic tests, and to prescribe and initiate treatment. They frequently pointed to the nature of SCI medicine, which requires management of multiple systems of the body and demands specialized skills in bladder, bowel, blood pressure, pain, spasticity, respiratory management and more, stating that they could not be “jack of all trades” (*Skills*). Related to the lack of skills in OSA diagnosis and treatment was the lack of confidence in their abilities to manage OSA. Several doctors reported diagnosing respiratory insufficiency and prescribing bi-level PAP treatment in the inpatient units without a respiratory physician, but they were not confident in diagnosing OSA or prescribing PAP for OSA (*Beliefs about capabilities)*.

The ability to diagnose and treat some respiratory disorders with bi-level PAP but not treat OSA with CPAP appeared to reflect historical management pathways and beliefs about professional responsibilities. A subset of those who reported referring to sleep specialists for diagnosis and treatment of OSA felt very strongly that OSA should only be managed by a sleep specialist; that it was outside of their scope of practice and that it would be irresponsible to take on management of OSA *(Social/Professional role and identity)*. For some this was also related to strict regulations from compensatory funding bodies. Seven of the doctors interviewed reported that funding bodies would only accept applications for PAP funding if the patient had been diagnosed with a full overnight polysomnography, and/or the diagnosis had been made by a sleep specialist. These regulations varied between and within countries (*Environmental context and resources*).

There were conflicting views about the benefit of PAP therapy. Those who were independently managing all aspects of non-complicated OSA were very positive about the benefits of PAP in terms of individual patient outcomes and overall adherence in their units. However many were disappointed in the treatment, reporting poor tolerance in the majority of their patients, which was frequently cited as a disincentive to screen for OSA (*Beliefs about consequences*).

Another commonly cited barrier was poor access to specialist sleep services. Several doctors described long waiting lists for overnight sleep studies and specialist consultations. For others the poor access was related to the inability of the sleep services to cater for the needs of people with disability. For example, the lack of nursing support provided by the sleep service and the lack of specialized equipment. However, some doctors were satisfied with their local specialist sleep services, describing good relationships and relatively short waiting times for appointments, and consequently reported no need to change their current practice of referring patients with suspected OSA (*Environmental context and resources*).

The availability (and lack) of allied health professionals and nurses with OSA management skills was both an enabler and a barrier to diagnosing and treating OSA within the spinal unit. The doctors who were performing any aspect of diagnosis or treatment in the inpatient or outpatient units reported their reliance on ancillary staff for support. The types of staff involved varied from unit to unit, but were usually nurses, physiotherapists or respiratory therapists. These staff tended to be involved in the application of diagnostic equipment (eg overnight oximetry or polygraphy), and/or treatment initiation and maintenance. Whilst doctors usually made the diagnosis and prescribed treatment, they mostly relied on the allied staff to perform the operational tasks. Conversely doctors not diagnosing or treating OSA tended to report the lack of available ancillary staff to support OSA management as a significant barrier to the practice (*Environmental context and resources*).

Almost every doctor interviewed reported similar OSA practices to his/her colleagues from the same spinal unit, pointing to culture and the local environment as highly significant (*Environmental context and resources*). The three doctors (and units) who were independently managing all aspects of non-complicated OSA spoke extensively of a highly influential “clinical champion” who introduced and led the OSA management program in their unit (*Social influences*).

Most doctors who were referring to specialists for OSA management thought that the diagnosis and treatment of non-complicated OSA could potentially be performed within their unit, provided there was additional training for staff *(Knowledge and skills)*, more resources for equipment and staff and changes to the funding rules for PAP devices (*Environmental context and resources*).

## Discussion

This is the first time the breadth of OSA management practices within a spinal unit has been investigated and documented, and the first time a behavioural model, such as the TDF, has been applied to this area of clinical medicine to explore the influences on clinical practice. We found that 40 to 50% of spinal doctors in our sample were undertaking routine screening for signs and symptoms of OSA in their patients with tetraplegia. The remainder reported being alerted to signs and symptoms from the patient, family or ward staff before any investigation for OSA. This reactive practice may have contributed to an under-diagnosis of OSA in this population. Most doctors in this study felt that routine screening for OSA was their responsibility and was a beneficial practice to either continue or initiate. A comparison of the influences on screening practices between those routinely screening and those who were not, found that time available in outpatients, resources for allied health and nursing support, and reminders to prompt screening were likely to be important.

Providing reminders is a common intervention to prompt clinicians to perform tasks such as screening for a condition or ordering an investigation. A 2012 overview of 35 systematic reviews of reminder interventions aiming to change clinical behaviours found that reminders can lead to modest improvements in clinical practice, and concluded they are an effective intervention across a range of healthcare settings [17]. If reminders for OSA screening were to be implemented in a spinal unit, consideration of the local context to determine the most suitable type of reminder would be important. Further research is needed to explore the feasibility and effectiveness of reminders as an intervention to improve rates of screening for OSA in spinal cord injury settings.

The types of screening varied significantly in the inpatient and outpatient environments, with objective tests (e.g. overnight oximetry) predominant in the inpatient unit, and questions about symptoms (e.g. daytime sleepiness, snoring) prevailing in the outpatient clinics. Two OSA screening questionnaires developed for the non-disabled sleep clinic population have been tested in the SCI and both performed poorly in identifying OSA, however their ability to identify individuals at high risk of OSA, who require objective testing has not been evaluated [3, 18]. Recently a two-stage model for identifying moderate to severe OSA in people with chronic tetraplegia has been validated and published [2]. In this model a screening questionnaire identifies patients who require further testing with overnight oximetry. This simple four-item questionnaire (SOSAT) could be applied in the outpatient setting of a spinal unit to identify high-risk individuals for further investigation.

Our group conducted a recent qualitative study, which sought to understand the experience of using CPAP to treat OSA among people with tetraplegia [19]. We found that people with tetraplegia tended not to recognise their symptoms of OSA until after they had been treated with CPAP and experienced the benefits. As a result, many did not report their symptoms to a health professional. Given this finding, along with the high prevalence of OSA, we would argue that spinal units should be routinely screening for OSA in tetraplegia [19].

Within our sample, three doctors reported that their spinal unit (15%) was predominantly managing all aspects of non-complicated OSA. Eleven (55%) described referring all patients with suspected OSA to sleep specialists for ongoing management, and six (30%) were performing some components of the diagnosis and/or treatment prescription, usually in the inpatient setting. Those referring to sleep specialists for OSA management tended to lack confidence and skills in interpreting diagnostic tests and prescribing treatments, and felt that OSA management was outside the scope of their specialty and should be managed by a sleep specialist. Seven (35% of the total sample) were also impeded by restrictive regulations from compensatory bodies that limit the diagnosis of OSA to sleep specialists. Spinal units independently managing non-complicated OSA in their patients were well resourced for staff and training, were not impeded by regulations from compensatory funding bodies, and described “clinical champions” who initiated and led the OSA program within their spinal unit. Most of the doctors who were not diagnosing and treating OSA thought that their unit could do so with additional training, equipment, and greater involvement of allied health professionals and/or nurses.

That almost half of the spinal doctors interviewed in this study were undertaking at least some diagnosis and/or treatment of non-complicated OSA suggests that it is entirely possible for spinal doctors to perform these tasks. The perception that OSA is outside of the scope of practice of a spinal doctor may be more likely to reflect local cultural influences, lack of training and resource constraints. The results of this study suggest that with adequate training and resources, spinal units that currently refer to sleep specialists for OSA management may be able to perform these practices within the unit. This is consistent with a non-randomised study in stroke survivors (reported to have a similarly high OSA prevalence to that observed in tetraplegia [20]) which demonstrated that it is feasible and safe to diagnose and treat OSA within a stroke rehabilitation environment [21].

Poor access and high costs of in-laboratory specialist sleep services to diagnose and initiate treatment for OSA have been identified as a problem in the non-disabled population [22, 23]. In response, alternative ambulatory techniques, including automated, home-based diagnosis and treatment initiation, have been compared to specialist sleep laboratory management, with all studies demonstrating non-inferiority of the alternative model [22, 24]. There have now been three non-inferiority randomised controlled trials investigating whether non-sleep specialist health professionals can effectively treat OSA in people without disability using these ambulatory techniques. Two investigated OSA management delivered in primary care settings by general practitioners and practice nurses [25, 26], with the other investigating OSA management provided by nurses in specialist sleep centres [27]. In each of the studies the alternative models were compared to the traditional sleep specialist model, and all concluded that the care provided by non-sleep specialist professionals was not inferior to that provided by the sleep specialists. As yet, there has been no research investigating alternatives to the specialist sleep model for people with tetraplegia.

Ideally, a randomised controlled trial comparing the spinal unit management of non-complicated OSA to specialist sleep laboratory management could determine whether spinal unit management is at least not inferior to the traditional model. The alternative OSA management model could be based on one, or a combination of the three, spinal units found to be independently managing OSA in this study. There are important safety and feasibility considerations, such as the identification and treatment of hypoventilation, to resolve prior to any such clinical trial. However evaluation of safety procedures at the spinal units identified in this study, and consultation with sleep specialists, should enable resolution of these concerns. In addition, our findings suggest that staff training, multi-disciplinary involvement, and resources for equipment are important components of the model. People with tetraplegia have previously identified overnight in-laboratory sleep studies as a major barrier to OSA diagnosis and subsequent treatment [19]. Managing OSA within the spinal unit could eliminate this known barrier and improve diagnosis rates.

### Limitations

Only five of the 20 interviews were independently double-coded in this study by two researchers (MG and DJB). However the coding framework was revised after the first five interviews and guided the analysis of the remaining 15, and any instances of ambiguity were discussed between the two researchers.

It is possible that the snowballing recruitment method resulted in the recruitment of participants with similar practices and beliefs, and thus saturation of themes could have occurred prematurely. However, participants were only asked to recommend doctors from different spinal units, and our purposive sampling method also involved recruiting participants from a range of countries in the OECD. The results demonstrate a wide variation in practice and beliefs. Self-reported clinical practice is also likely to be influenced by these sampling techniques and the small sample size. Whilst we are confident that our matrix of clinical practices describes the range of OSA practices in the OECD, we do not suggest that the proportions of doctors allocated to the different clinical practices in this study can be generalised to all spinal doctors in the OECD.

Interviewing clinicians about their perceived influences on their clinical practice does not necessarily reveal the actual influences on their practices [28]. Triangulation is a commonly used technique in qualitative research, involving the use of multiple data sources to facilitate deeper understanding. Ideally the findings of this study should be compared and complimented with a quantitative clinical practice audit and, given the multi-disciplinary nature of OSA management, more qualitative research involving spinal unit nurses, allied health clinicians and people with tetraplegia.

## Conclusion

People with tetraplegia experience high disability and disadvantage. In this context, while we recognise that knowledge translation interventions should be primarily focused on clinical areas with robust evidence-based recommendations for clinical practice, we are advocating for the translation of best available evidence into practice. We assert that routine screening for a highly prevalent condition, for which there is a relatively cheap, simple and non-invasive treatment available, is both practical and worthwhile. Given the lack of specific, actionable practice recommendations in the existing guidelines, and the wide variation in OSA management practices described in this study, more research into the feasibility and outcomes of spinal unit management of non-complicated OSA is warranted. Interventions that target the factors identified in this study are likely to improve the management of OSA, which may ultimately improve the quality of life of people living with tetraplegia.

## Additional file

Additional file 1: This supplementary file contains the interview guide used in the study. (DOCX 38 kb)

## Data Availability

Not applicable. Data are transcripts of qualitative interviews and are therefore potentially identifying. Participants did not consent for their interview data to be publicly available.

## Abbreviations

CPAP: Continuous Positive Airway Pressure
OECD: Organisation for Economic Co-operation and Development
OSA: Obstructive Sleep Apnoea
PAP: Positive airway pressure
SCI: Spinal Cord Injury
SCIRE: Spinal cord injury rehabilitation evidence
TDF: Theoretical Domains Framework
