# Supplementary material for: Understanding the clinical management of obstructive sleep apnoea in tetraplegia: a qualitative study using the theoretical domains framework
Source: BMC Health Serv Res. 2019 Jun 21;19:405. doi: 10.1186/s12913-019-4197-8 (PMC6588842; doi:10.1186/s12913-019-4197-8)
Supplement: Supplementary file 1 — This supplementary file contains the interview guide used in the study. (DOCX 38 kb) [file 12913_2019_4197_MOESM1_ESM.docx]

**TITLE:**

Understanding the clinical management of obstructive sleep apnoea in tetraplegia: a qualitative study using the theoretical domains framework.

**AUTHORS:** Marnie Graco^1 2^, David J Berlowitz ^1 3^, Sally E Green^4^

*Institutions:*

^1^ Institute for Breathing and Sleep, Austin Health, Melbourne, Victoria, Australia

^2^ The University of Melbourne, Department of Medicine, Melbourne, Victoria, Australia

^3^ The University of Melbourne, Department of Physiotherapy, Melbourne, Victoria, Australia

^4^ Monash University, School of Public Health and Preventive Medicine, Melbourne, Victoria, Australia

**Additional file 1: Interview guide for doctors managing patients with tetraplegia.**

**Introduction**

As outlined in the participant information sheet, this study aims to identify and explore the factors that influence the management of tetraplegic patients with obstructive sleep apnoea.

You do not have to answer every question and can cease the interview at any time. If you need to attend to an urgent matter we can stop the interview and recommence it later.

We will talk about your spinal unit, how you screen patients for obstructive sleep apnoea, how you manage patients with suspected or confirmed obstructive sleep apnoea, and the factors that influence this management. We are interviewing spinal physicians from around the world, and in different sized units, with and without access to specialist sleep services so that we get a broad view. Interviews will be audio-taped and you will be provided with a copy of the transcript and invited to provide feedback on the accuracy of the information. All information will be treated as strictly confidential.

Before we start do you have any questions?

**Background information**

Can you describe your spinal unit, its clientele and the services it offers?

Prompt questions

- How many years of experience do you have working as a spinal physician?
- How many inpatient beds do you have?
- What is the mix of traumatic vs non-traumatic injuries?
- How many new patients do you admit per year?
- What outpatient/ outreach services do you have?
- How big is the hospital?
  - Emergency department? Trauma services?
- How would you describe the socio-economic status of your patient population?
- Is there a sleep laboratory in the hospital? What sleep services do they offer? If not, where are the nearest specialist sleep services? Is there a waiting list for services?

Now I am going to ask you questions about how you manage OSA in your SCI patients, and what factors influence your clinical practice. First I will ask how and why you screen for and diagnose OSA in both the inpatient and outpatient units, and then we will ask about how and why you manage a positive diagnosis of OSA.

**Screening for and diagnosing obstructive sleep apnoea**

**Understanding clinical practice**

Can you talk me through the various steps in how you screen patients for obstructive sleep apnoea in:

- The inpatient unit
- The outpatient clinic

Prompt questions:

- Do you screen patients for signs and symptoms of OSA?
  - If so, how do you screen? What questions do you ask? Which signs and symptoms alert you to possible OSA?
  - Are there any particular clinical signs or symptoms that you would consider as high risk? How would you measure these?
  - Who does the screening?
  - Do you routinely screen everyone? If not, who do you (not) screen?
  - When do you screen? At what time point post injury? Roughly what proportion of your SCI outpatients do you screen for signs and symptoms? Is this different for paraplegia/quadriplegia?
- Do you order diagnostic tests for suspected OSA?
  - If so, what tests do you order? If more than one type, how would you decide which test?
  - Who orders the testing?
  - Who performs the testing?
  - Which patients do you test for OSA?
  - Roughly what proportion of your SCI outpatients do you refer for testing for OSA? Is this different for paraplegia/quadriplegia?
  - Where are the results of the assessments/tests recorded? (and by who?)
  - How are the results used? (and by who?)
  - How do you determine whether a patient has a diagnosis of OSA?
  - Do you know roughly how long your patients wait for diagnostic test?

**Factors influencing practice**

Now I want to ask you about what influences your OSA screening practices. I am using a framework called the Theoretical Domains Framework, which is a set of 12 domains that are known to influence clinical behaviours. Some of the questions will seem more relevant than others.

Firstly, before I prompt with specific questions about potential factors that are known to influence clinical practice, can I first ask you tell me what you think are the biggest influences on your decision to screen/not screen your patients for OSA in both the inpatient and outpatient settings?

Prompt questions to explore factors influencing practice (grouped by TDF domains).

| **TDF Domains** | **TDF Definitions [Constructs]** | **Prompt questions (if required)** |
| --- | --- | --- |
| Knowledge | An awareness of the existence of something. [Knowledge including knowledge of condition/scientific rationale. Procedural knowledge. Knowledge of task environment.] | Are you aware of any clinical practice guidelines recommending screening for OSA?  What do you think is best practice in management of OSA in tetraplegia?  Are you aware of any research about the prevalence and impact of OSA in SCI?  Are you familiar with any risk assessment tools? |
| Skills | An ability or proficiency acquired through practice. [Skills Skills development Competence Ability Interpersonal skills Practice Skill assessment] | What skills are needed?  Do you know how to order a diagnostic test for OSA?  Do you know how to interpret results of screening and diagnostic tests for OSA? |
| Social professional role and identity | A coherent set of behaviors and displayed personal qualities of an individual in a social or work setting. [Professional identity Professional role Social identity Identity Professional boundaries Professional confidence Group identity Leadership Organizational commitment] | Do you think screening/diagnosing OSA is part of your role as physician? If not, whose role is it?  Is there a commitment from your organization to manage OSA in tetraplegia? |
| Beliefs about capabilities | Acceptance of the truth, reality, or validity about an ability, talent, or facility that a person can put to constructive use. [Self-confidence Perceived competence Self-efficacy Perceived behavioral control Beliefs Self-esteem Empowerment Professional confidence] | Any difficulties in assessing signs and symptoms of OSA?  Any challenges in determining presence of OSA in general and using/ordering different tests/tools in particular?  What would help you to identify your patients with OSA?  How confident are you that you can identify OSA in your patients? |
| Beliefs about consequences | Acceptance of the truth, reality, or validity about outcomes of a behaviour in a given situation. [Beliefs Outcome expectancies Characteristics of outcome expectancies Anticipated regret Consequences] | What do you think are the benefits and costs of screening for OSA in people with tetraplegia? (for your patients, you, your colleagues and the organization)  What are the benefits and costs of not screening for OSA? (for your patients, you, your colleagues and the organization)  What will happen if you don’t routinely screen?  Do the benefits outweigh the costs?  Does the evidence suggest that screening is worthwhile? |
| Motivation and goals | A conscious decision to perform a behavior or resolve to act in a certain way. Mental representations of outcomes or end states that an individual wants to achieve. [Stability of intentions Stages of change model Transtheoretical model and stages of change Goals (distal/proximal) Goal priority Goal/target setting Goals (autonomous/controlled) Action planning Implementation intention] | Are there incentives to screen for OSA?  Do you feel you have to?  Are there other aspects of your role that interfere with screening for OSA? |
| Memory, attention and decision processes | The ability to retain information, focus selectively on aspects of the environment and choose between two or more alternatives. [Memory Attention Attention control Decision making Cognitive overload/tiredness] | Are there any reminders in place to prompt you to do any of the relevant tests? If no, do you think these would be helpful?’  Is it something you do routinely?  Is screening for OSA something you do if you have time? |
| Environmental context and resources | Any circumstance of a person’s situation or environment that discourages or encourages the development of skills and abilities, independence, social competence, and adaptive behavior. [Environmental stressors Resources/material resources Organizational culture/climate Salient events/critical incidents Person x environment interaction Barriers and facilitators] | Do resources influence whether you assess these patients for OSA?  Are there sufficient human resources?  Are there sufficient physical resources?  Do you have enough time/do you have competing demands?  Does the working environment in inpatient/ outpatients have an effect?  Are there environmental stressors that impact on your ability to screen for OSA?  Do rules/regulations from compensation bodies ever influence your decisions about screening/diagnosing OSA? |
| Social influences | Those interpersonal processes that can cause individuals to change their thoughts, feelings, or behaviors. [Social pressure Social norms Group conformity Social comparisons Group norms Social support Power Intergroup conflict Alienation Group identity Modeling] | Do you seek opinions of colleagues in whether to screen for OSA/ interpreting test results?  What are the views of your colleagues re: screening for OSA?  Do you observe others screening patients for OSA? |
| Emotion | A complex reaction pattern, involving experiential, behavioral, and psychological elements, by which an individual attempts to deal with a personally significant matter or event. [Fear  Anxiety Affect Stress Depression Positive/negative effect Burn-out] | Is there anything about screening for OSA that evokes an emotional response? If so, what? Does this alter your clinical management decisions? |
| Behavioral regulation | Anything aimed at managing or changing objectively observed or measured actions. [Self-monitoring Breaking habit Action planning] | Are there any protocols or referral pathways that facilitate screening for OSA? |

**Treatment of obstructive sleep apnoea**

**Understanding clinical practice**

Can you talk me through the various steps in how you treat patients with a diagnosis of obstructive sleep apnoea in both the inpatient and outpatient units?

Prompt questions

- Are all patients diagnosed with OSA offered treatment?
  - If not, under what circumstances would they not be referred for treatment?
- Who provides the treatment?
- What is the process for referring a person with OSA for treatment?
- Where is the referral for treatment recorded?
- What treatment/s are offered to patients with OSA?
- How is the decision to offer a particular treatment made?
- Would you/others involve the patient in the decision? If so, how?
- Where are the details of the treatment prescribed and the outcome of the treatment recorded?
- If a particular treatment is not successful (ie not accepted by the patient), what happens next? Are they referred for an alternative treatment?
- Do you know roughly how long your patients wait for treatment?

**Factors influencing practice**

Now I want to ask you about what influences your OSA treatment practices. Firstly, before I prompt with specific questions about potential factors that are known to influence clinical practice, can I first ask you tell me what you think are the biggest influences on your decisions to treat your patients for OSA in both the inpatient and outpatient settings?

Prompt questions to explore factors influencing practice (grouped by TDF domains).

| **TDF Domains** | **TDF Definitions [Constructs][25]** | **Prompt questions (if required)** |
| --- | --- | --- |
| Knowledge | An awareness of the existence of something. [Knowledge including knowledge of condition/scientific rationale. Procedural knowledge. Knowledge of task environment.] | Are you aware of any clinical practice recommendations regarding treatment of OSA in tetraplegia?  What do you think is best practice in management of OSA in tetraplegia?  Are you aware of any research about the effectiveness of OSA treatments in SCI? |
| Skills | An ability or proficiency acquired through practice. [Skills Skills development Competence Ability Interpersonal skills Practice Skill assessment] | What skills are needed to treat OSA?  Do you know how to prescribe treatment for OSA?  Do you know how to initiate treatment for OSA? |
| Social professional role and identity | A coherent set of behaviors and displayed personal qualities of an individual in a social or work setting. [Professional identity Professional role Social identity Identity Professional boundaries Professional confidence Group identity Leadership Organizational commitment] | Do you think prescribing treatment for OSA is part of your role as spinal physician? If not, whose role is it?  Do you think it would ever be feasible for OSA treatment to become the role of the spinal physician or spinal unit? If not, why?  Is there a commitment from your organization to manage OSA in tetraplegia? |
| Beliefs about capabilities | Acceptance of the truth, reality, or validity about an ability, talent, or facility that a person can put to constructive use. [Self-confidence Perceived competence Self-efficacy Perceived behavioral control Beliefs Self-esteem Empowerment Professional confidence] | Any difficulties in prescribing treatment for OSA?  What would help you to effectively treat your patients with OSA?  How confident are you that you can effectively treat OSA in your patients? |
| Beliefs about consequences | Acceptance of the truth, reality, or validity about outcomes of a behaviour in a given situation. [Beliefs Outcome expectancies Characteristics of outcome expectancies Anticipated regret Consequences] | What are the benefits and costs of treating OSA in people with tetraplegia? (for your patients, you, your colleagues and the organization)  What are the benefits and costs of not treating OSA? (for your patients, you, your colleagues and the organization)  What will happen if you don’t prescribe treatment?  Do the benefits outweigh the costs?  Does the evidence suggest that treatment for OSA is worthwhile? |
| Motivation and goals | A conscious decision to perform a behavior or resolve to act in a certain way. Mental representations of outcomes or end states that an individual wants to achieve. [Stability of intentions Stages of change model Transtheoretical model and stages of change Goals (distal/proximal) Goal priority Goal/target setting Goals (autonomous/controlled) Action planning Implementation intention] | Are there incentives to treat OSA?  Do you feel you have to?  Are there other aspects of your role that interfere with treating OSA? |
| Memory, attention and decision processes | The ability to retain information, focus selectively on aspects of the environment and choose between two or more alternatives. [Memory Attention Attention control Decision making Cognitive overload/tiredness] | Are there any reminders in place to prompt you to prescribe/refer for treatment? If no, do you think these would be helpful?’  Is it something you do routinely? |
| Environmental context and resources | Any circumstance of a person’s situation or environment that discourages or encourages the development of skills and abilities, independence, social competence, and adaptive behavior. [Environmental stressors Resources/material resources Organizational culture/climate Salient events/critical incidents Person x environment interaction Barriers and facilitators] | Do resources influence whether you prescribe/refer patients for OSA treatment?  Are there sufficient human resources?  Are there clear communication channels?  Are there sufficient physical resources?  Do you have enough time/do you have competing demands?  Does the working environment in outpatients have an effect?  Are there environmental stressors that impact on your ability to treat OSA? |
| Social influences | Those interpersonal processes that can cause individuals to change their thoughts, feelings, or behaviors. [Social pressure Social norms Group conformity Social comparisons Group norms Social support Power Intergroup conflict Alienation Group identity Modeling] | Do you seek opinions of colleagues in whether/how to treat OSA?  What are the views of your colleagues re: OSA treatment?  Do you observe others treating patients for OSA? |
| Emotion | A complex reaction pattern, involving experiential, behavioral, and psychological elements, by which an individual attempts to deal with a personally significant matter or event. [Fear  Anxiety Affect Stress Depression Positive/negative effect Burn-out] | Is there anything about prescribing/referring for OSA treatment that evokes an emotional response? If so, what? Does this alter your clinical management decisions? |
| Behavioral regulation | Anything aimed at managing or changing objectively observed or measured actions. [Self-monitoring Breaking habit Action planning] | Are there any protocols or referral pathways that facilitate OSA treatment? |

If time permits and only if these items have not yet been covered:

- What do you think are the key actions/decisions when managing a patient with OSA that maximize the beneficial outcomes for the patient?
- Is there an aspect of the patient pathway we should pay more attention to in future interviews?
- If there was one thing you could change in your spinal unit to improve the management of OSA in people with tetraplegia, what would you change?
- Do you think it would ever be feasible for your spinal unit to take on the diagnosis and treatment of non-complicated? If yes, what would be required to do this? If not, why?

Final questions:

- Is there anything else about the management of patients with OSA that you would like to mention that is not already covered?
- Do you have any additional comments on the content of the interview or feedback on how the interview went?

**THANK YOU VERY MUCH FOR YOUR TIME**
